# Supplementary material for: One-step fermentative production of aromatic polyesters from glucose by metabolically engineered Escherichia coli strains
Source: Nat Commun. 2018 Jan 8;9:79. doi: 10.1038/s41467-017-02498-w (PMC5758686; doi:10.1038/s41467-017-02498-w)
Supplement: Supplementary file 1 — Supplementary Information [file 41467_2017_2498_MOESM1_ESM.doc]

**SUPPLEMENTARY INFORMATION**


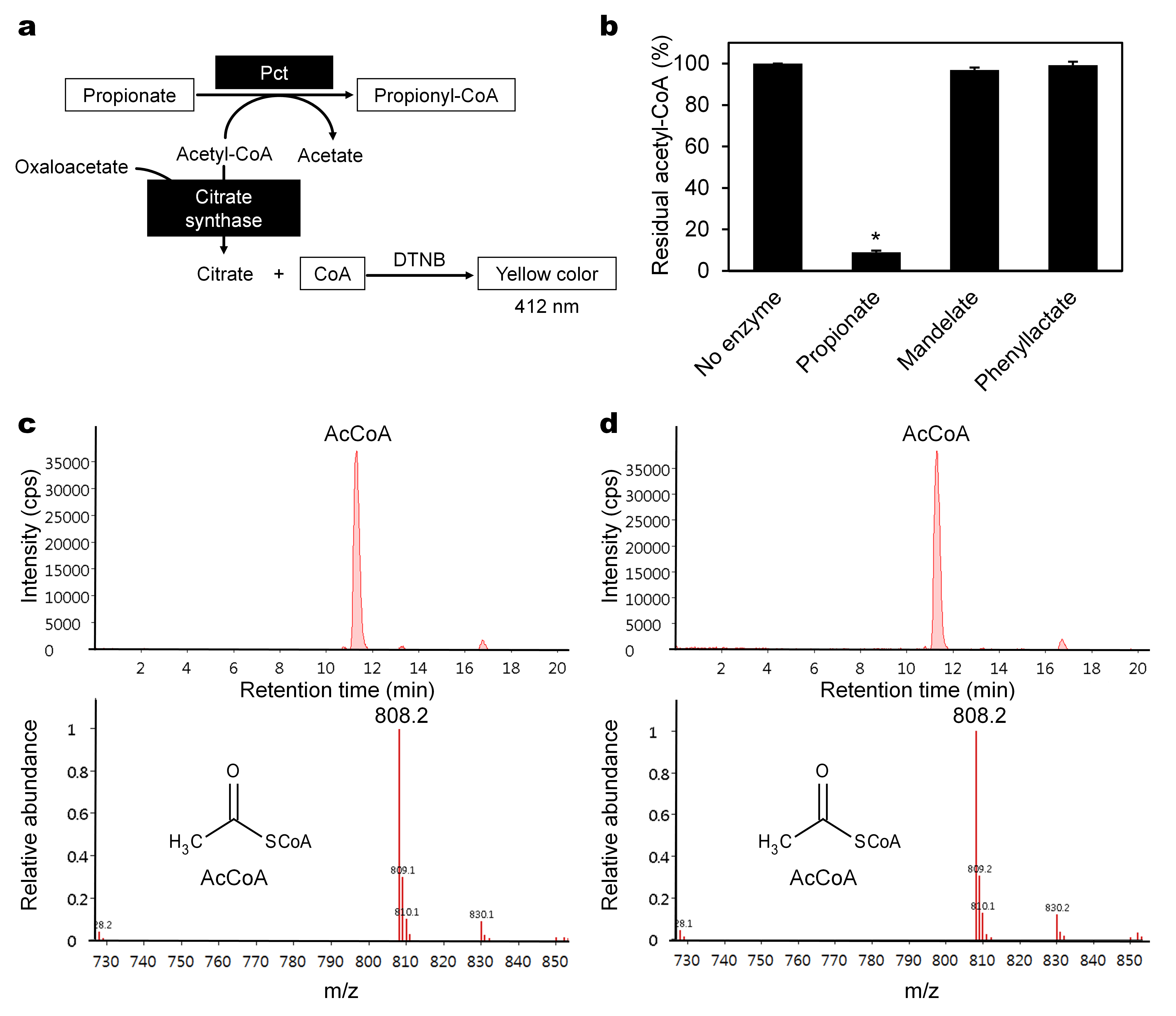


**Supplementary Figure 1. *In vitro* assay of Pct540.** **a**, Scheme of reaction used in the assay. **b**, Analysis of residual amount of acetyl-CoA at the end point of *in vitro* assay (*n* = 3 each). **P* < 0.05 by two-tailed *t*-test in comparison to control (No enzyme). All data are mean ± s.d. **c-d**, LC-MS analysis of CoA derivatives extracted from *in vitro* reaction mixture using mandelate (**b**) or phenyllactate (**c**) as a substrate. The LC-MS samples were prepared by extraction of CoA thioesters at the end point of *in vitro* Pct540 enzyme assay as described in Methods.


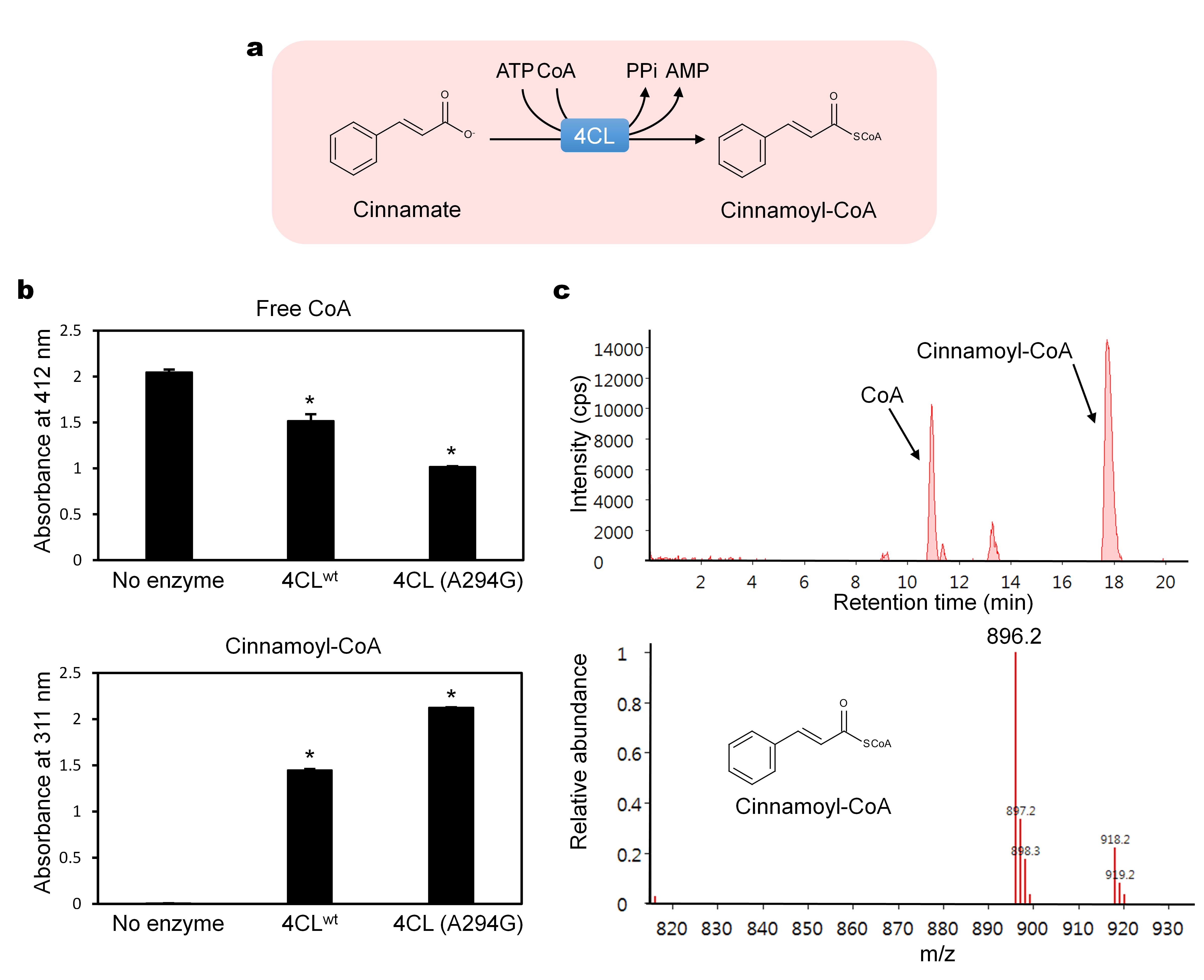
**Supplementary Figure 2.** ***In vitro* enzyme assay of 4CL and 4CL(A294G).** **a**, Scheme of reaction used in the assay. **b**, Analysis of residual amount of free CoA and cinnamoyl-CoA at the end point of *in vitro* assay (*n* = 3 each). **P* < 0.05 by two-tailed *t*-test in comparison to control (No enzyme). Error bars represent ± s.d.. **c**, LC-MS analysis of cinnamoyl-CoA generated by 4CL(A294G). The monosodium cinnamoyl-CoA was also detected. The LC-MS sample is prepared by extraction of CoA derivatives from reaction mixture as described in Methods. The LC-MS samples were prepared by extraction of CoA thioesters at the end point of *in vitro* 4CL(A294G) enzyme assay as described in Methods.


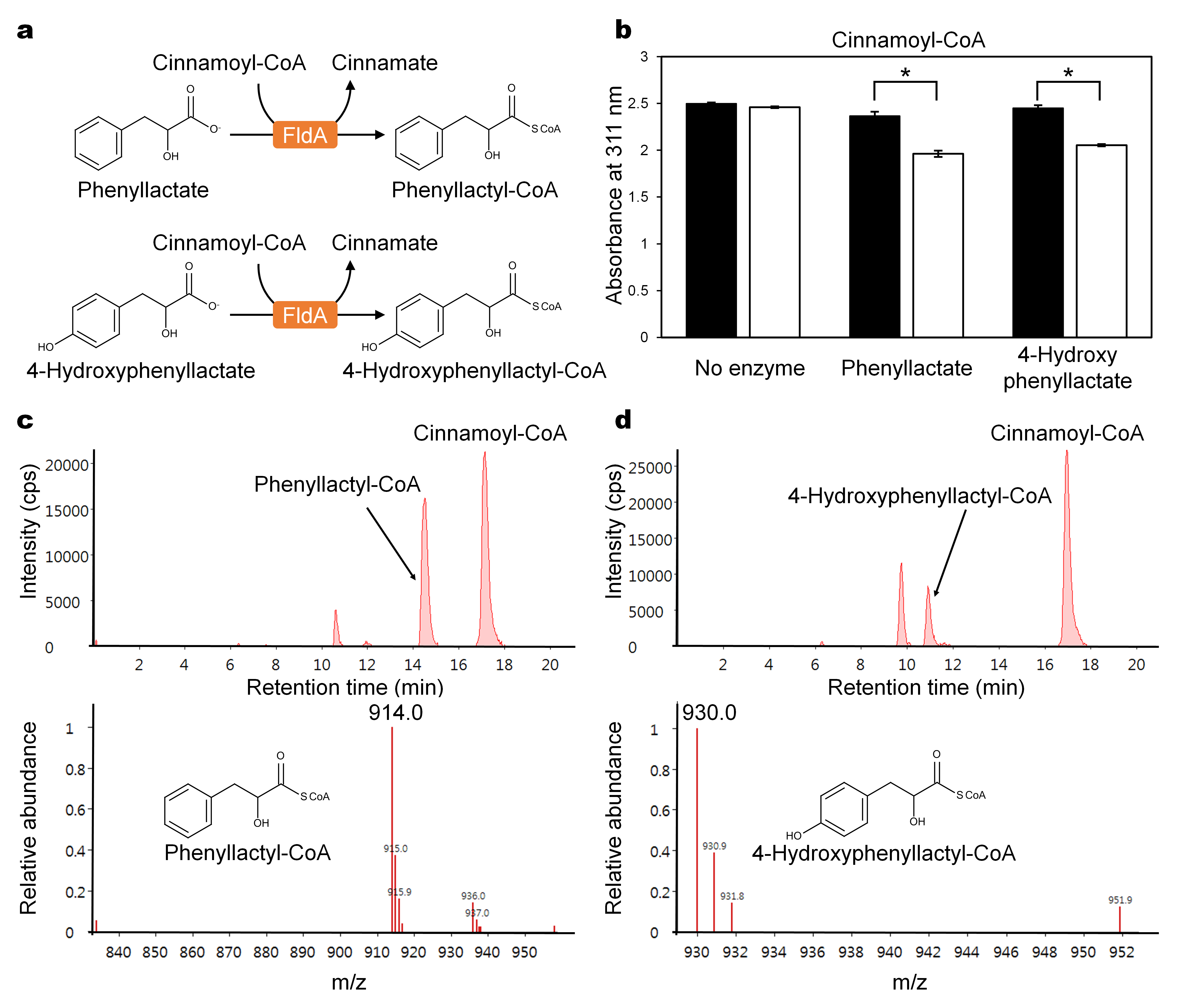


**Supplementary Figure 3. *In vitro* enzyme assay of FldA.** **a**, Scheme of reactions used in the assay. **b**, Analysis of remaining cinnamoyl-CoA at the end point of *in vitro* enzyme assay. **c-d**, LC-MS analysis of phenyllactyl-CoA (**c**) and 4-hydroxyphenyllactyl-CoA (**d**) generated by FldA. The monosodium phenyllactyl-CoA and monosodium 4-hydroxyphenyllactyl-CoA were also detected. **P* < 0.05, by two-tailed *t*-test. Error bars represent s.d. of *n*=3 technical replicates. The LC-MS samples were prepared by extraction of CoA thioesters at the end point of *in vitro* FldA enzyme assay as described in Methods.


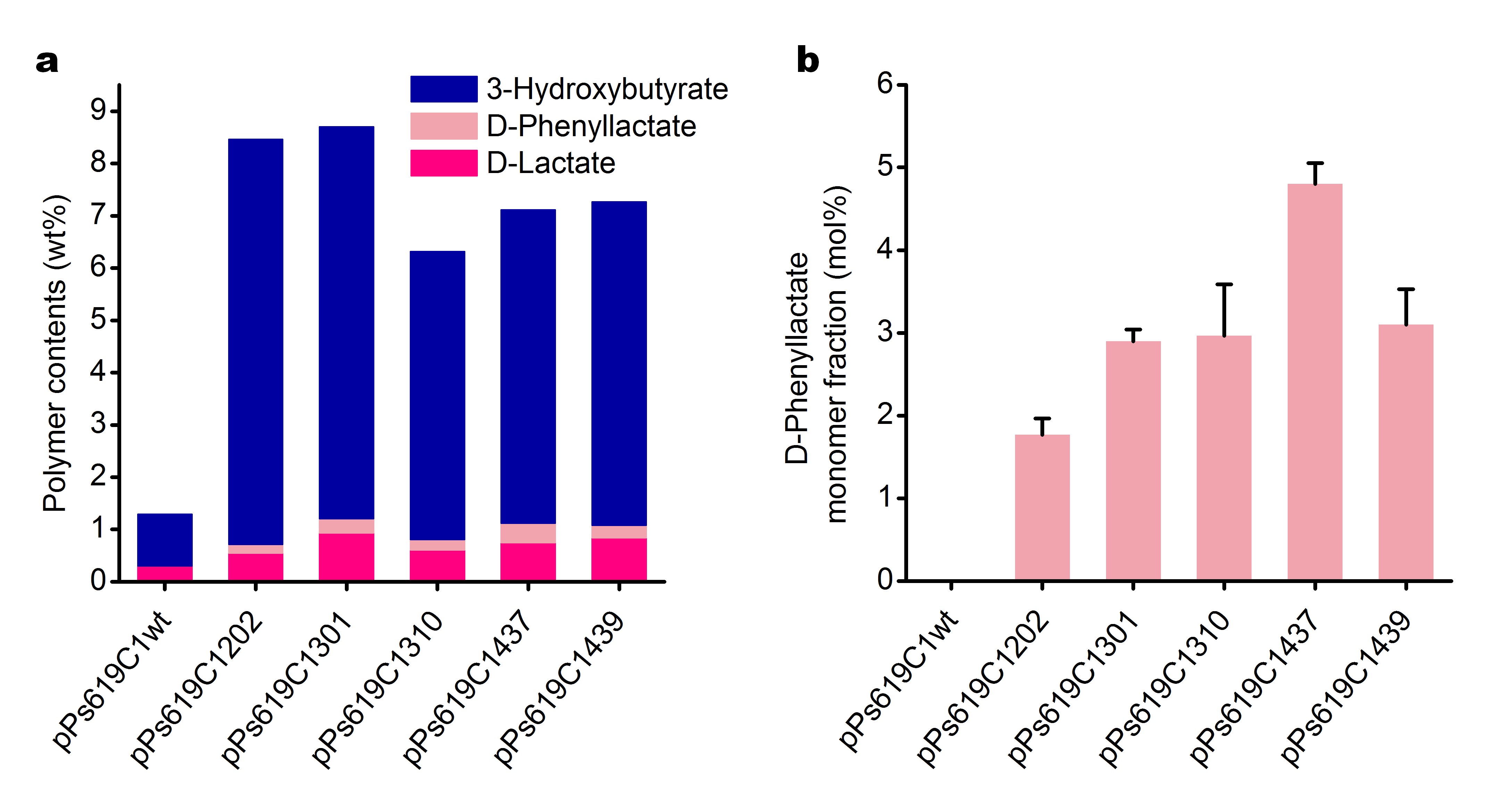


**Supplementary Figure 4.** **Aromatic PHA production using *Pseudomonas* sp. MBEL 6-19 PHA synthase variants.** **a-b**, Analysis of polymer contents, compositions (**a**) and d-phenyllactate monomer fraction (**b**). Error bars represent s.d. of *n*=3 technical replicates.

**
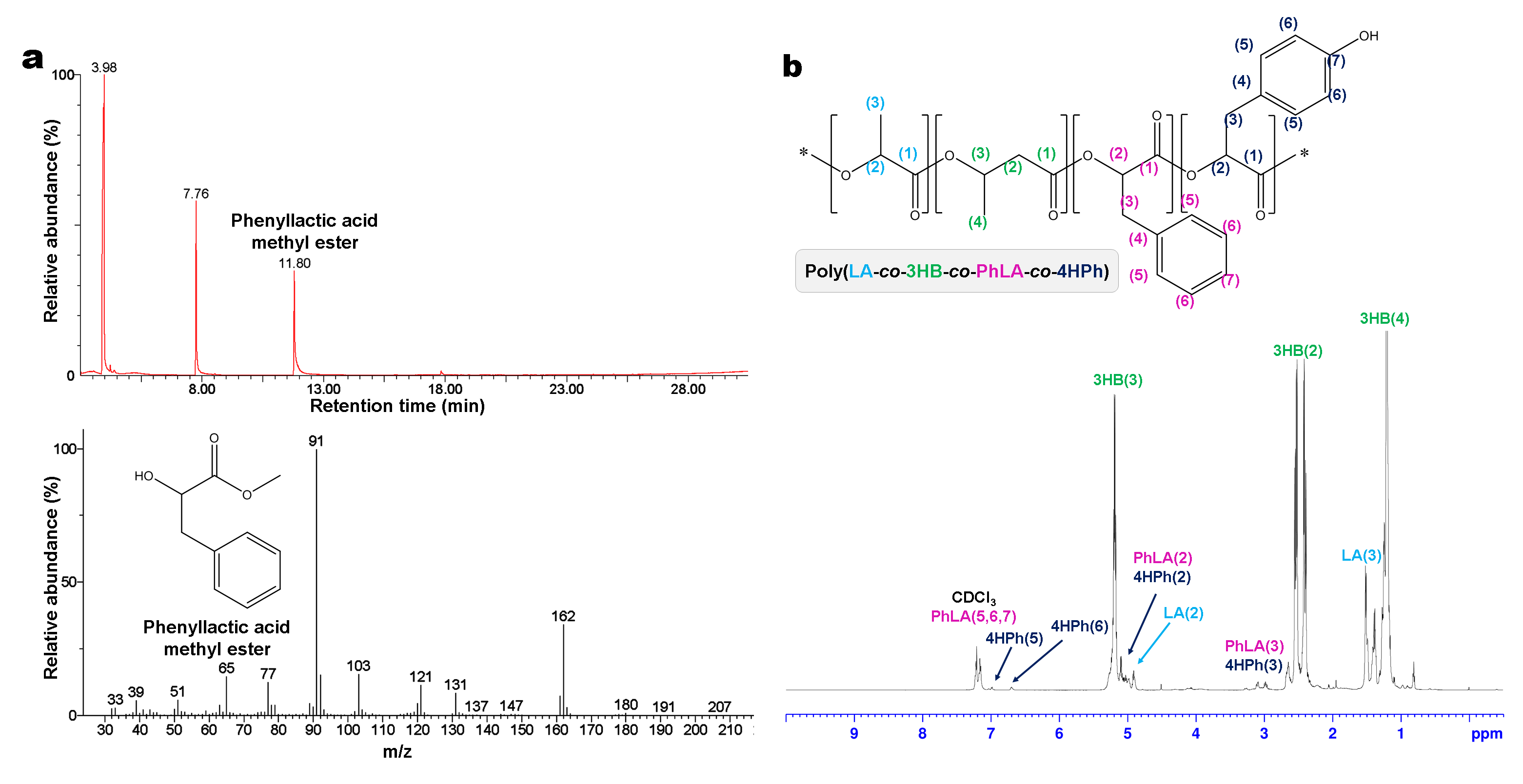
**

**Supplementary Figure 5. GC-MS and ^1^H NMR** **analysis of polymer produced by FldA.** **a**, The GC-MS analysis of methylated d-phenyllactate prepared by methylation of the polymer produced in *E. coli* XL1-Blue expressing AroG^fbr^, FldH together with overexpression of PAL, 4CL, FldA, Pct540 and PhaC1437 from glucose. **b**, ^1^H NMR analysis of poly(d-lactate-*co*-3HB-*co*-d-phenyllactate-*co*-d-4-hydroxyphenyllactate).

**
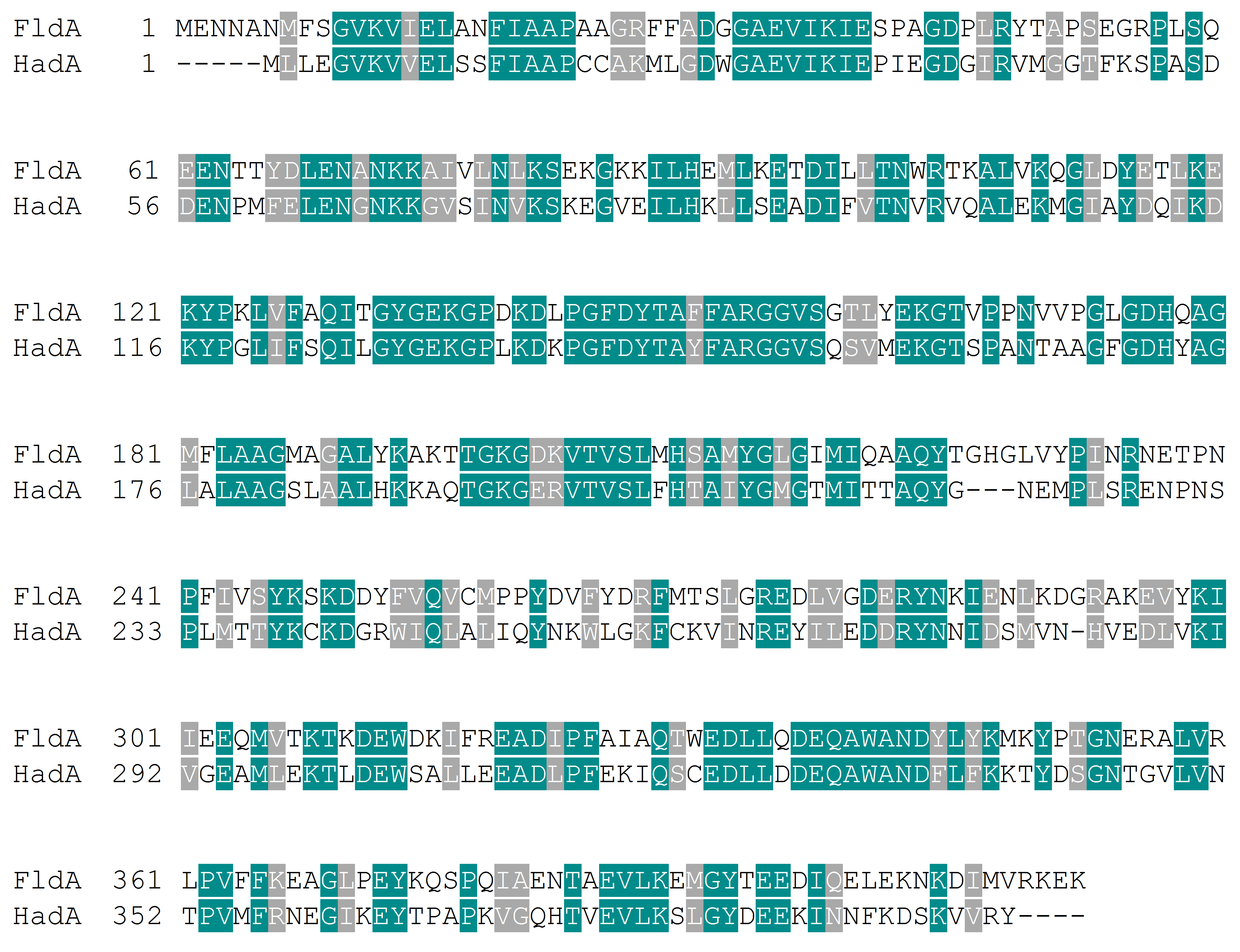
**

**Supplementary Figure 6. Amino acid sequence alignment of *Clostridium botulinum* FldA and *Clostridium difficile* HadA.** The sequence identity and similarity were indicated by blue green and light gray colors, respectively.

**
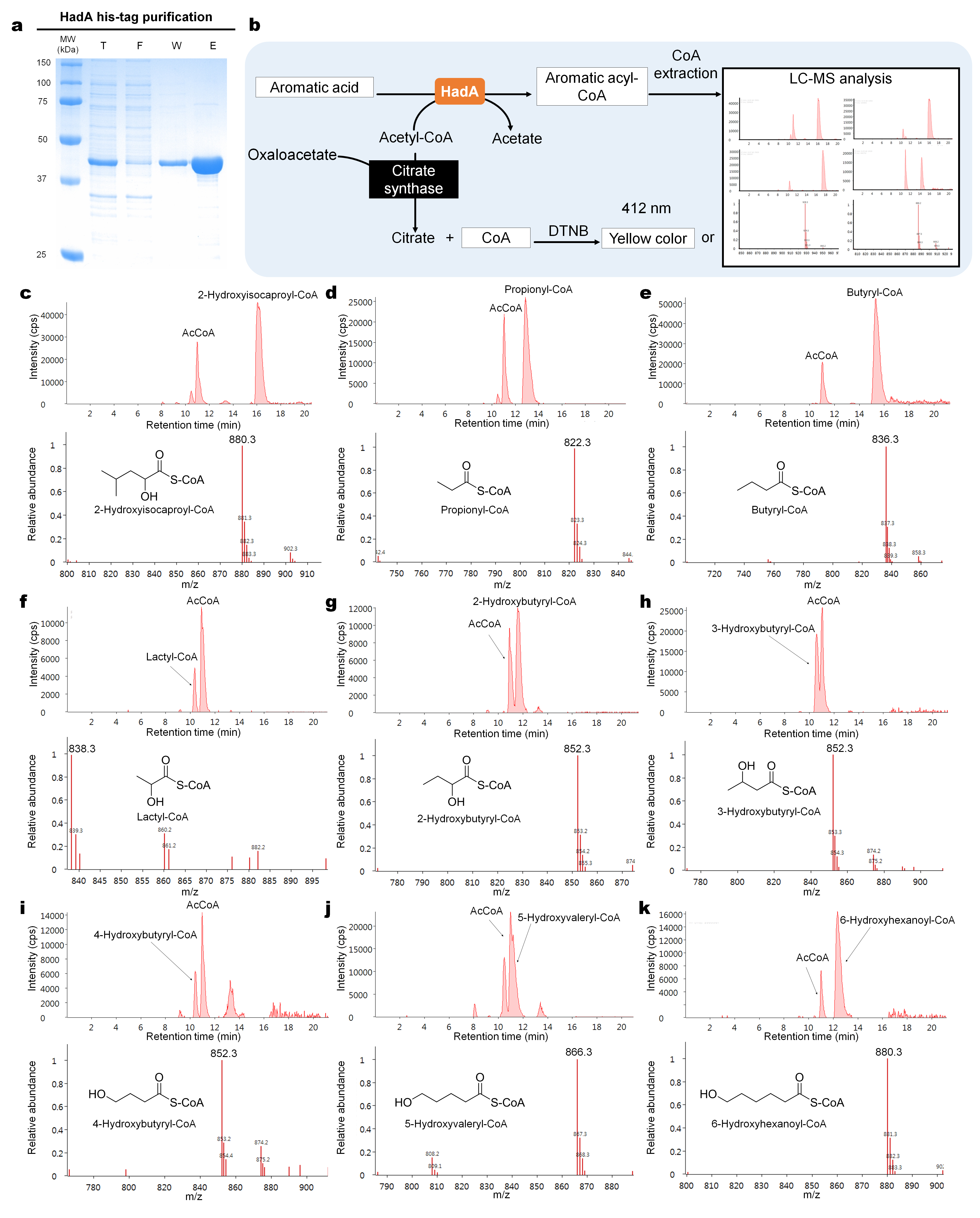
**

**Supplementary Figure 7. *In vitro* enzyme assay of HadA and LC-MS analysis of various CoA thioesters generated by HadA.** **a**, SDS-PAGE analysis of purified his-tagged HadA. The lanes are: T, total proteins; F, unbound protein fraction; W, washed fraction; E, eluted fraction; Mw, molecular weight marker (kDa). **b**, Schematic reaction of the assay. LC-MS analysis of 2-hydroxyisocaproyl-CoA (**c**), propionyl-CoA (**d**), butyryl-CoA (**e**), lactyl-CoA (**f**), 2-hydroxybutyryl-CoA (**g**), 3-hydroxybutyryl-CoA (**h**), 4-hydroxybutyryl-CoA (**i**), 5-hydroxyvaleryl-CoA (**j**), and 6-hydroxyhexanoyl-CoA (**k**) from reaction mixture. The LC-MS samples were prepared by extraction of CoA thioesters at the end point of *in vitro* HadA enzyme assay as described in Methods.

**
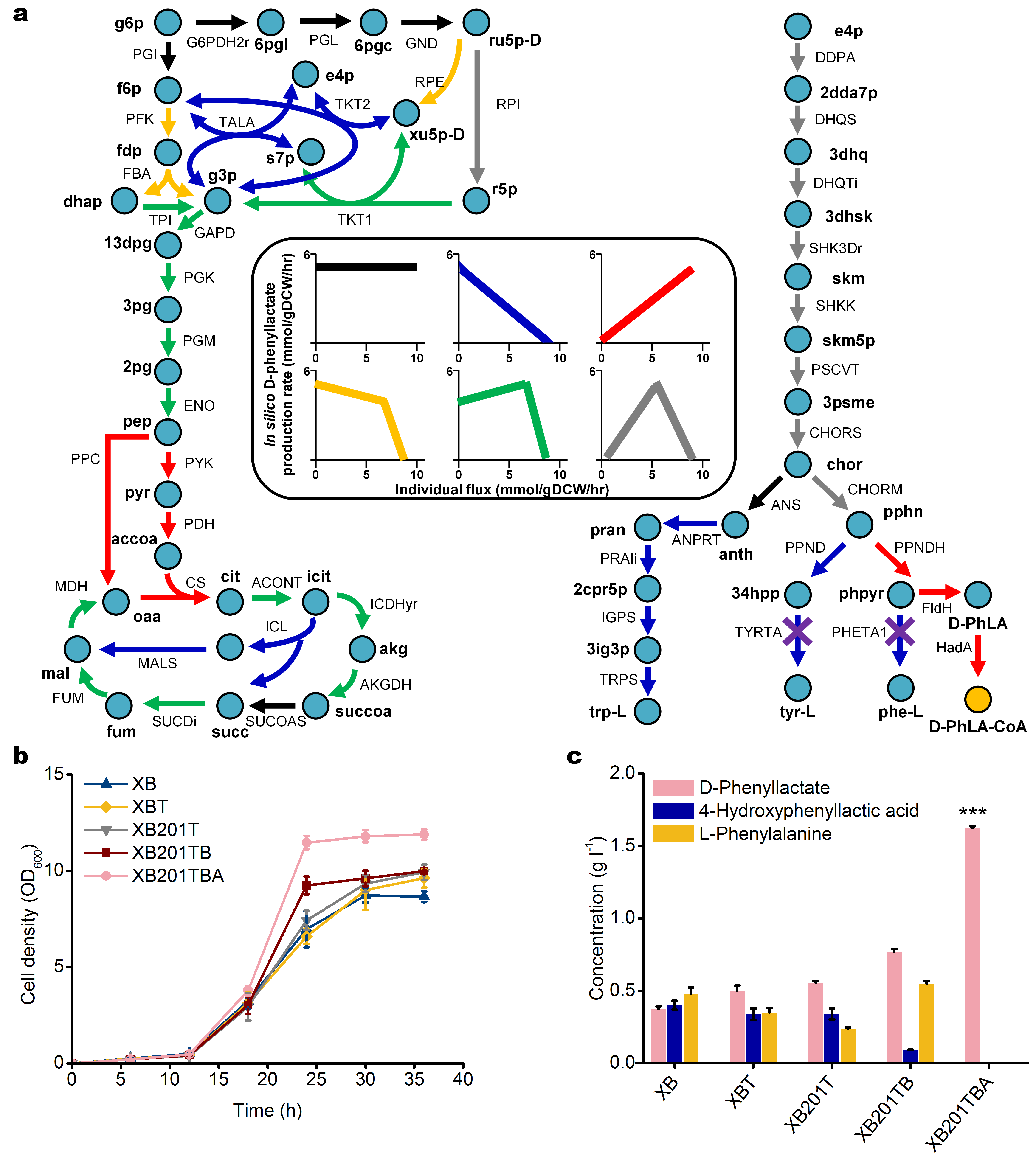
**

**Supplementary Figure 8. *In silico* genome-scale simulation driven metabolic engineering.** **a**, *In silico* genome-scale metabolic flux analysis. The responses of the d-phenyllactic acid production rate to fluxes of central and aromatic amino acid biosynthesis reactions were analyzed. The response results of each reaction were divided into six types and represented as colors of the arrows. Those reactions that negatively related with d-phenyllactic acid production rate (i.e. blue and yellow) were identified as potential knockout candidates. The purple Xs indicate that the deletion of the corresponding gene based on simulation results. Abbreviations are described in Supplementary Information. **b**, The time profiles of cell growth (OD_600_). **c**, Analysis of phenyllactic acid, 4-hydroxyphenyllactic acid and l-phenylalanine produced by engineered *E. coli* strains harboring pKM212-AroG^fbr^pheA^fbr^ and pACYC-FldH. ****P* < 0.001, by two-tailed *t*-test in comparison to XB. Error bars represent s.d. of *n*=3 technical replicates. Abbreviations for reactions are as follows: ACONT, aconitase; AKGDH, 2-oxogluterate dehydrogenase; ANPRT, anthranilate phosphoribosyltransferase; ANS, anthranilate synthase; CHORM, chorismate mutase; CHORS, chorismate synthase; CS, citrate synthase; DDPA, 3-deoxy-d-arabino-heptulosonate 7-phosphate synthetase; DHQS, 3-dehydroquinate synthase; DHQTi, 3-dehydroquinate dehydratase; ENO, enolase; FBA, fructose-bisphosphate aldolase; FUM, fumarase; G6PDH2r, glucose-6-phosphate dehydrogenase; GAPD, glyceraldehyde-3-phosphate dehydrogenase; GND, phosphogluconate dehydrogenase; ICDHyr, isocitrate dehydrogenase; ICL, isocitrate lyase; IGPS, indole-3-glycerol-phosphate synthase; MALS, malate synthase; MDH, malate dehydrogenase; PDH, pyruvate dehydrogenase; PFK, phosphofructokinase; PGI, glucose-6-phosphate isomerase; PGK, phosphoglycerate kinase; PGL, 6-phosphogluconolactonase; PGM, phosphoglycerate mutase; PHETA1, phenylalanine transaminase; PPC, phosphoenolpyruvate carboxylase; PPND, prephenate dehydrogenase; PPNDH, prephenate dehydratase; PRAIi, phosphoribosylanthranilate isomerase; PRPPS, phosphoribosylpyrophosphate synthetase; PSCVT, 3-phosphoshikimate 1-carboxyvinyltransferase; PYK, pyruvate kinase; RPE, ribulose-5-phosphate 3-epimerase; RPI, ribose-5-phosphate isomerase; SHK3Dr, shikimate dehydrogenase; SHKK, shikimate kinase; SUCDi, succinate dehydrogenase; SUCOAS, succinyl-CoA synthetase; TALA, transaldolase; TKT1, transketolase; TKT2, transketolase; TPI, triose-phosphate isomerase; TRPS, tryptophan synthase; TYRTA, tyrosine transaminase. Abbreviations for metabolites are as follows: 13dpg, 3-Phospho-d-glyceroyl phosphate; 2cpr5p, 1-(2-Carboxyphenylamino)-1-deoxy-d-ribulose 5-phosphate; 2dda7p, 2-Dehydro-3-deoxy-d-arabino-heptonate 7-phosphate; 2pg, d-Glycerate 2-phosphate; 34hpp, 4-Hydroxyphenylpyruvate; 3dhq, 3-Dehydroquinate; 3dhsk, 3-Dehydroshikimate; 3ig3p, C'-(3-Indolyl)-glycerol 3-phosphate; 3pg, 3-Phospho-d-glycerate; 3psme, 5-O-(1-Carboxyvinyl)-3-phosphoshikimate; 6pgc, 6-Phospho-d-gluconate; 6pgl, 6-phospho-d-glucono-1,5-lactone; accoa, Acetyl-CoA; akg, 2-Oxoglutarate; anth, Anthranilate; chor, chorismate; cit, Citrate; dhap, Dihydroxyacetone phosphate; d-PhLA-CoA, d-Phenyllactyl-CoA; e4p, d-Erythrose 4-phosphate; f6p, d-Fructose 6-phosphate; fdp, d-Fructose 1,6-bisphosphate; fum, Fumarate; g3p, Glyceraldehyde 3-phosphate; g6p, d-Glucose 6-phosphate; glx, Glyoxylate; icit, Isocitrate; mal, l-Malate; oaa, Oxaloacetate; pep, Phosphoenolpyruvate; phe-l, l-Phenylalanine; phpyr, Phenylpyruvate; pphn, Prephenate; pran, N-(5-Phospho-d-ribosyl)anthranilate; prpp, 5-Phospho-alpha-d-ribose 1-diphosphate; pyr, Pyruvate; r5p, alpha-d--Ribose 5-phosphate; ru5p-d, d-Ribulose 5-phosphate; s7p, Sedoheptulose 7-phosphate; skm, Shikimate; skm5p, Shikimate 5-phosphate; succ, Succinate; succoa, Succinyl-CoA; trp-l, l-Tryptophan; tyr-l, l-Tyrosine; xu5p-d, d-Xylulose 5-phosphate.


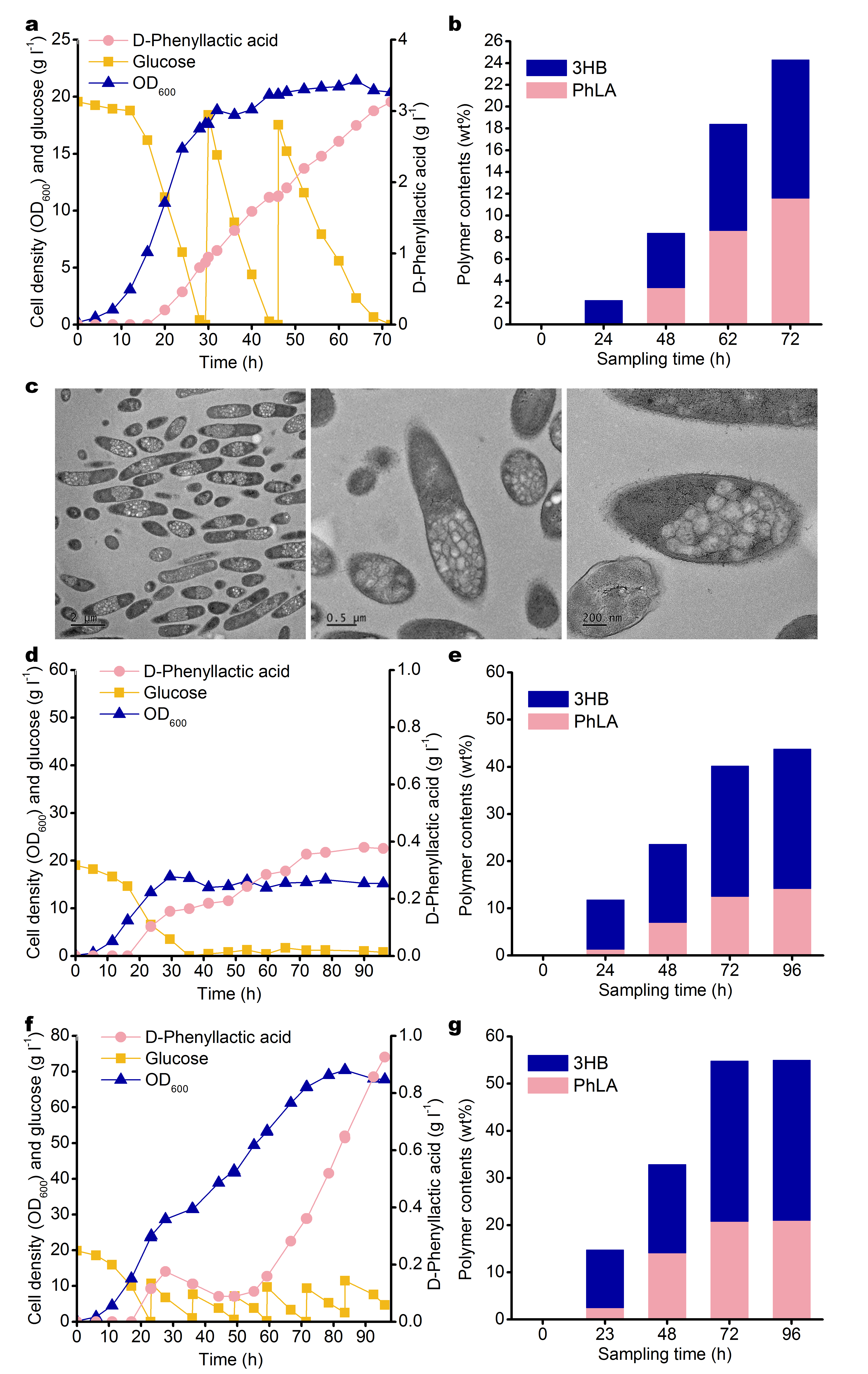


**Supplementary Figure 9. Fed-batch fermentation profiles.** (**a**) The time profiles of cell growth (OD_600_) and production of d-phenyllactic acid from glucose and sodium 3HB, (**b**) polymer contents and compositions by pulsed-feeding (to make glucose concentration to 20 g l^-1^ and sodium 3HB concentration to 1 g l^-1^) fed-batch culture of *E. coli* XB201TBAL expressing AroG^fbr^, PheA^fbr^, FldH, HadA and PhaC1437. (**c**) the transmission electron micrography (TEM) image of *E. coli* XB201TBAL expressing AroG^fbr^, PheA^fbr^, FldH, HadA and PhaC1437. Scale bars, 2 μm, 0.5 μm, and 200 nm. (**d**) The time profiles of cell growth and production of d-phenyllactic acid from only glucose, and (**e**) polymer contents and compositions by pH-stat fed-batch culture of *E. coli* XB201TBAL strain expressing AroG^fbr^, PheA^fbr^, FldH, HadA, PhaC1437 and PhaAB under BBa_J23114 promoter. (**f**) The time profiles of cell growth and production of d-phenyllactic acid from only glucose, and (**g**) polymer contents and compositions by pulsed-feeding (to make glucose concentration to 10 g l^-1^) fed-batch culture of *E. coli* XB201TBAF strain expressing AroG^fbr^, PheA^fbr^, FldH, HadA, PhaC1437 and PhaAB under BBa_J23114 promoter.


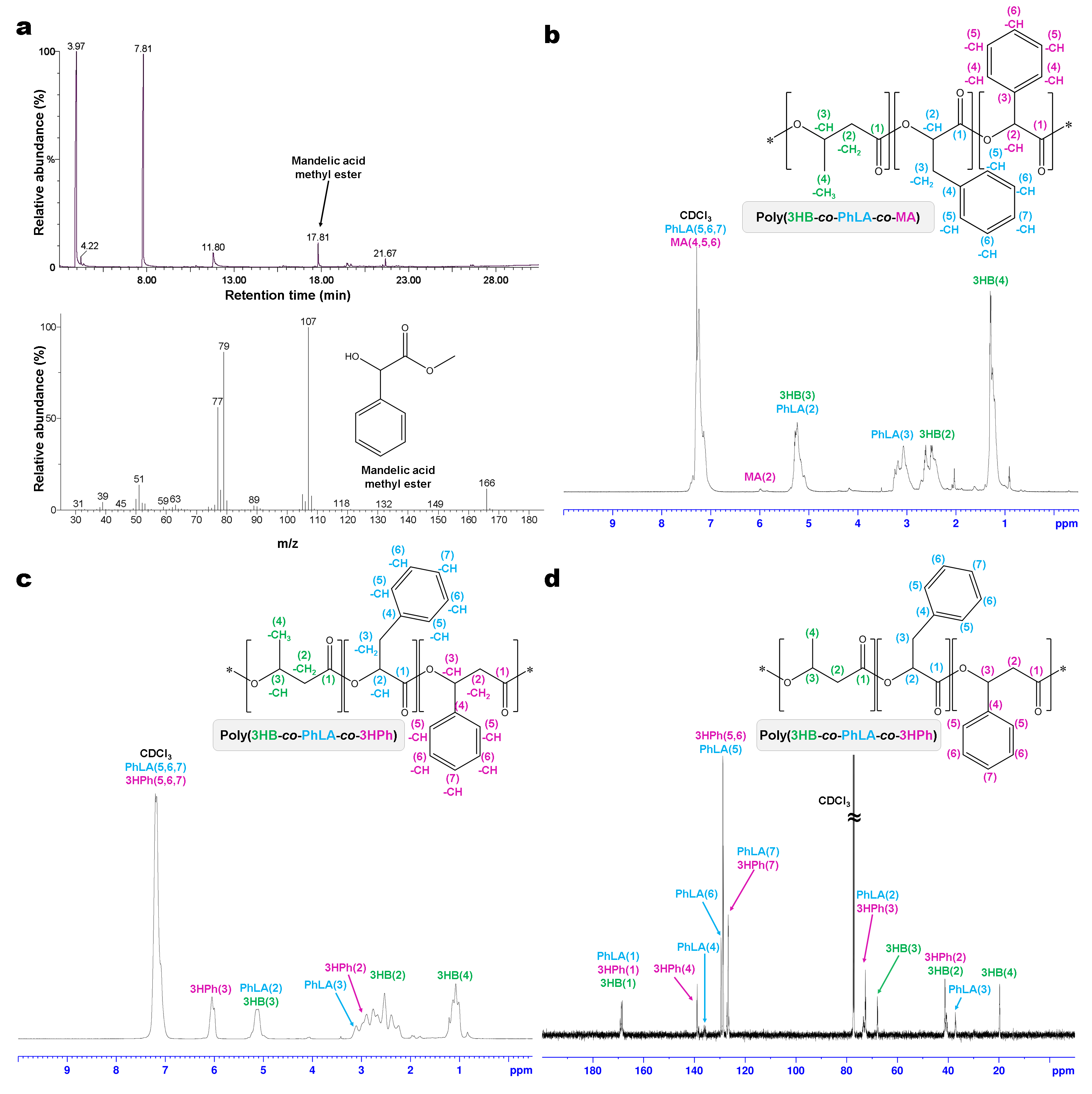


**Supplementary Figure 10.** **NMR analysis of poly(3HB-*co*-d-phenyllactate-*co*-d-mandelate) and poly(3HB-*co*-d-phenyllactate-*co*-d-3-hydroxy-3-phenylpropionate).** GC-MS analysis (**a**) and ^1^H NMR (**b**) of poly(3HB-*co*-d-phenyllactate-*co*-d-mandelate) produced by *E. coli* XL1-Blue strain expressing AroG^fbr^, PheA^fbr^, FldH, HadA and PhaC1437 cultured in chemically defined medium containing 0.5 g l^-1^ d-mandelic acid and 1 g l^-1^ sodium 3HB. ^1^H NMR (**c**) and ^13^C NMR spectra (**d**) of poly(3HB-*co*-d-phenyllactate-*co*-d-3-hydroxy-3-phenylpropionate) produced by *E. coli* XL1-Blue strain expressing AroG^fbr^, PheA^fbr^, FldH, HadA and PhaC1437 cultured in chemically defined medium containing 0.5 g l^-1^ d-3-hydroxy-3-phenylpropionic acid and 1 g l^-1^ sodium 3HB.

**Supplementary Table 1.** Properties of PHAs produced by metabolically engineered *E. coli*.

| **Strain** | **Plasmids** | **Mole fraction (mol% ± s.d.)** | | | |  | **Molecular weight (Da)** | | |  | **T_g_ (ºC)** |  | **T_c_ (ºC)** |  | **T_m_ (ºC)** |  | **ΔH_m_ (J/g)** |
| --- | --- | --- | --- | --- | --- | --- | --- | --- | --- | --- | --- | --- | --- | --- | --- | --- | --- |
|  |  | **LA** | **3HB** | **PhLA** | **4HPLA** |  | **M_n_** | **M_w_** | **M_w_/M_n_** |  |  |  |  |  |  |  |  |
| *E. coli* XL1-Blue^a^ | pPs619C1437-HadA | 0 | 100 | 0 | 0 |  | 31373 | 46418 | 1.48 |  | -3.13 |  | 40.79 |  | 164.73 |  | 61.67 |
| *E. coli* XL1-Blue^b^ | pPs619C1437-HadA | 16.8 ± 0.4 | 80.8 ± 0.6 | 1.6 ± 0.1 | 0.8 ± 0.1 |  | 15769 | 25169 | 1.60 |  | -3.41 |  | 79.70 |  | 146.59 |  | 26.03 |
|  | pKM212-AroG^fbr^PAL |  |  |  |  |  |  |  |  |  |  |  |  |  |  |  |  |
|  | pACYC-4CL(A294G)FldAH |  |  |  |  |  |  |  |  |  |  |  |  |  |  |  |  |
| XB201TB^c^ | pPs619C1437-HadA | 0 | 64.5 ± 0.2 | 35.5 ± 0.1 | 0 |  | 7444 | 11645 | 1.56 |  | 7.12 |  | c |  | 153.33 |  | 4.89 |
|  | pKM212-AroG^fbr^PheA^fbr^ |  |  |  |  |  |  |  |  |  |  |  |  |  |  |  |  |
|  | pACYC-FldH |  |  |  |  |  |  |  |  |  |  |  |  |  |  |  |  |
| XB201TBAL^c^ | pPs619C1437-HadA | 0 | 52.1 ± 0.4 | 47.9 ± 0.2 | 0 |  | 4757 | 6264 | 1.32 |  | 23.86 |  | - |  | - |  | - |
|  | pKM212-AroG^fbr^PheA^fbr^ |  |  |  |  |  |  |  |  |  |  |  |  |  |  |  |  |
|  | pACYC-FldH |  |  |  |  |  |  |  |  |  |  |  |  |  |  |  |  |

^a^For poly(3HB) production, the *E. coli* XL1-Blue harboring pPs619C1437-HadA was cultured under the supplementation of 1 g l^-1^ sodium 3-hydroxybutyrate. ^b^For poly(d-lactate-*co*-3HB-*co*-d-phenyllactate-*co*-d-4-hydroxyphenyllactate) production, the *E. coli* XL1-Blue harboring pKM212-AroG^fbr^PAL, pACYC-4CL(A294G)FldAH and pPs619C1437-HadA was used. ^c^Each strain pKM212-AroG^fbr^PheA^fbr^, pACYC-FldH and pPs619C1437-HadA was cultured. ^d^Broad T_c_ ranging from 80-110 was observed. LA, 3HB, PhLA and 4HPLA represent d-lactate, 3-hydroxybutyrate, d-phenyllactate and d-4-hydroxyphenyllactate, respectively. Data represent mean ± s.d. (*n*=3 technical replicates).

**Supplementary Table 2.** Characterization of aromatic PHAs with different monomer fractions.

| **Synthetic promoters**^a^ | **Mole fraction (mol% ± s.d.)** | |  | **Molecular weight (Da)** | | |  | **T_g_ (ºC)**^b^ |
| --- | --- | --- | --- | --- | --- | --- | --- | --- |
|  | **3HB** | **PhLA** |  | **M_n_** | **M_w_** | **M_w_/M_n_** |  |  |
| BBa_J23100 | 89.0 ± 0.8 | 11.0 ± 0.5 |  | 24920 | 50120 | 2.01 |  | 9.41 |
| BBa_J23105 | 84.2 ± 2.7 | 15.8 ± 0.4 |  | 22470 | 45460 | 2.02 |  | 10.05 |
| BBa_J23114 | 80.0 ± 1.8 | 20.0 ± 1.3 |  | 15760 | 25850 | 1.64 |  | 15.64 |
| BBa_J23109 | 29.2 ± 1.1 | 70.8 ± 1.2 |  | 2665 | 4184 | 1.57 |  | 29.04 |
| BBa_J23103 | 15.5 ± 0.6 | 84.5 ± 3.3 |  | 3569 | 4588 | 1.29 |  | 33.47 |

^a^For the production of aromatic PHAs with different d-phenyllactate monomer fractions, XB201TBAL strain expressing PhaAB under synthetic Anderson promoters (BBa_J23100, BBa_J23105, BBa_J23114, BBa_J23109, or BBa_J23103) followed by overexpression of AroG^fbr^, PheA^fbr^, FldH, HadA and PhaC1437 were cultivated. ^b^The absence of T_m_ is a result of the amorphous polymer properties. 3HB and PhLA represent 3-hydroxybutyrate and d-phenyllactate, respectively. Data represent mean ± s.d. (*n*=3 technical replicates).

**Supplementary Table 3. Strains and plasmids used in this study**

| **Strain or plasmid** | **Relevant characteristics*** Abbreviations: Ap, ampicillin; Km, kanamycin; Cm, chloramphenicol; ^R^, resistance. | **Reference** |
| --- | --- | --- |
| XL1-Blue | *recA1 endA1 gyrA96 thi-1 hsdR17 supE44 relA1 lac* [F′ *proAB lacI^q^ZΔM15* Tn*10* (Tet^R^)] | Stratagene^a^ |
| BL21(DE3) | BL21(DE3) *F*− *ompT hsdSB (rB*−*mB*−*) gal dcm (DE3)* | Invitrogen^b^ |
| XBT | XL1-Blue Δ*tyrR* | This study |
| XB201T | XL1-Blue Δ*tyrR* △*poxB* △*pflB* △*adhE* △*frdB* | This study |
| XB201TB | XL1-Blue Δ*tyrR* △*poxB* △*pflB* △*adhE* △*frdB* Δ*tyrB* | This study |
| XB201TBA | XL1-Blue Δ*tyrR* △*poxB* △*pflB* △*adhE* △*frdB* Δ*tyrB* Δ*aspC* | This study |
| XB201TBAL | XL1-Blue Δ*tyrR* △*poxB* △*pflB* △*adhE* △*frdB* Δ*tyrB* Δ*aspC* Δ*ldhA* | This study |
| XB201TBAF | XL1-Blue Δ*tyrR* △*poxB* △*pflB* △*adhE* △*frdB* Δ*tyrB* Δ*aspC* *ΔldhA::Ptrc-fldH-rrnBT* | This study |
| pKD46 | λ-Red recombinase under arabinose inducible *araBAD* promoter, temperature sensitive origin; Ap^R^ | 1 |
| pJW168 | lox66-cat-lox71 cassette; Cm^R^ Ap^R^ | 2 |
| pMloxC | Cre-recombinase under IPTG inducible *lac*UV5 promoter, temperature sensitive origin; Ap^R^ | 3 |
| pMtrc9 | pMloxC derivative; *trc* promoter downstream of lox66-cat-lox71 cassette; Ap^R^ | Lab stock |
| pKM212-MCS | pBBR1MCS2 derivative; tac promoter, *Ralstonia* eutropha PHA biosynthesis genes transcription terminator; Km^R^ | 4 |
| pET-22b(+) | Expression vector, *T7* promoter; Ap^R^ | Novagen^c^ |
| pPs619C1437Pct540 | pBluescript II KS(+) derivative; *R. eutropha* PHA biosynthesis operon promoter, *Pseudomonas* sp. MBEL 6-19 *phaC_P_*_s6-19_ variant (*phaC1437*; E130D, S325T, S477G, Q481K), *Clostridium propionicum pct_Cp_* variant (*pct540*; V193A, silent mutations: T78C, T669C, A1125G, T1158C), transcriptional terminator of the *R. eutropha* PHA biosynthesis operon; Ap^R^ | 5 |
| pPs619C1wtPct540 | pPs619C1437Pct540 derivative; *phaC1437* was replaced by *phaC1_Ps_*_6-19_ wild type; Ap^R^ | 6 |
| pPs619C1202Pct540 | pPs619C1437Pct540 derivative; *phaC1437* was replaced by *phaC1202* (E130D,Q481K); Ap^R^ | 5 |
| pPs619C1301Pct540 | pPs619C1437Pct540 derivative; *phaC1437* was replaced by *phaC1301* (E130D,S325T, Q481K); Ap^R^ | 5 |
| pPs619C1310Pct540 | pPs619C1437Pct540 derivative; *phaC1437* was replaced by *phaC1310* (E130D, S477F, Q481K); Ap^R^ | 5 |
| pPs619C1439Pct540 | pPs619C1437Pct540 derivative; *phaC1437* was replaced by *phaC1439* (E130D, S325T, S477F, Q481K); Ap^R^ | 5 |
| pCnCAB | pBluescript II KS(+) derivative; *R. eutropha* PHA biosynthesis operon promoter, *R. eutropha phaCAB* genes, transcriptional terminator of the *R. eutropha* PHA biosynthesis operon, Ap^R^ | 5 |
| pCnAB | pCnCAB derivative; *R. eutropha* PHA biosynthesis operon promoter, *R. eutropha phaAB*; Ap^R^ | 7 |
| pPs619C1437-HadA | pBluescript II KS(+) derivative; *R. eutropha* PHA biosynthesis operon promoter, *Pseudomonas* sp. MBEL 6-19 *phaC_P_*_s6-19_ variant (*phaC1437*; E130D, S325T, S477G, Q481K), *Clostridium difficile hadA* gene, transcriptional terminator of the *R. eutropha* PHA biosynthesis operon; Ap^R^ | This study |
| pET22b-hisPCT540 | pET22b(+) derivative; *T7* promoter, the *C. propionicum* *pct540* gene; Ap^R^ | This study |
| pET22b-his4CL | pET22b(+) derivative; ***T7*** promoter, the *Streptomyces coelicolor* 4CL gene; Ap^R^ | This study |
| pET22b-his4CL(A294G) | pET22b(+) derivative; ***T7*** promoter, the *S. coelicolor* 4CL(A294G) gene; Ap^R^ | This study |
| pET22b-hisFldA | pET22b(+) derivative; ***T7*** promoter, the *Clostridium botulinum* A str. ATCC 3502 *fldA* gene; Ap^R^ | This study |
| pKM212-AroG^fbr^ | pKM212-MCS derivative; *tac* promoter, the *E. coli* feedback resistant *aroG*(D146N) gene; Km^R^ | This study |
| pKM212-AroG^fbr^PAL | pKM212-MCS derivative; *tac* promoter, the *E. coli* feedback resistant *aroG*(D146N) and *Streptomyces maritimus* *PAL* genes; Km^R^ | This study |
| pKM212-AroG^fbr^PheA^fbr^ | pKM212-MCS derivative; *tac* promoter, the *E. coli* feedback resistant *aroG*(D146N) and *pheA*(T326P) genes; Km^R^ | This study |
| pKM212-GPE-PhaAB | pKM212-MCS derivative; *tac* promoter, the *E. coli* feedback resistant *aroG*(D146N)*, pheA*(T326P)*, R. eutropha* PHA biosynthesis operon promoter, *R. eutropha phaA and phaB* genes; Km^R^ | This study |
| pKM212-GPE-100PhaAB | pKM212-MCS derivative; *tac* promoter, the *E. coli* feedback resistant *aroG*(D146N)*, pheA*(T326P)*,* BBa_J23100 promoter, *R. eutropha phaA and phaB* genes; Km^R^ | This study |
| pKM212-GPE-105PhaAB | pKM212-MCS derivative; *tac* promoter, the *E. coli* feedback resistant *aroG*(D146N)*, pheA*(T326P)*,* BBa_J23105 promoter, *R. eutropha phaA and phaB* genes; Km^R^ | This study |
| pKM212-GPE-114PhaAB | pKM212-MCS derivative; *tac* promoter, the *E. coli* feedback resistant *aroG*(D146N)*, pheA*(T326P)*,* BBa_J23114 promoter, *R. eutropha phaA and phaB* genes; Km^R^ | This study |
| pKM212-GPE-109PhaAB | pKM212-MCS derivative; *tac* promoter, the *E. coli* feedback resistant *aroG*(D146N)*, pheA*(T326P)*,* BBa_J23109 promoter, *R. eutropha phaA and phaB* genes; Km^R^ | This study |
| pKM212-GPE-103PhaAB | pKM212-MCS derivative; *tac* promoter, the *E. coli* feedback resistant *aroG*(D146N)*, pheA*(T326P)*,* BBa_J23103 promoter, *R. eutropha phaA and phaB* genes; Km^R^ | This study |
| pTrc-FldH | pTrc99A derivative; *trc* promoter, the *C. botulinum* A str. ATCC 3502 *fldH* gene; Ap^R^ | This study |
| pACYC184KS | pACYC184 derivative; MCS of pBluescript II KS in *Xba*I and *Nae*I site of pACYC184; Cm^R^ | This study |
| pACYC-FldH | pACYC184KS derivative; *trc* promoter, the *C. botulinum* A str. ATCC 3502 *fldH* gene; Cm^R^ | This study |
| pACYC-4CL(A294G) | pACYC184KS derivative; *trc* promoter, the *S. coelicolor* 4CL(A294G) gene; Cm^R^ | This study |
| pACYC-4CL(A294G)FldA | pACYC184KS derivative; *trc* promoter, the *S. coelicolor* 4CL(A294G) and the *C. botulinum* A str. ATCC 3502 *fldA* genes; Cm^R^ | This study |
| pACYC-4CL(A294G)FldAH | pACYC184KS derivative; *trc* promoter, the *S. coelicolor* 4CL(A294G), the *C. botulinum* A str. ATCC 3502 *fldA* and *fldH* genes; Cm^R^ | This study |
| pET22b-hisHadA | pET22b(+) derivative; ***T7*** promoter, the *C. difficile* A str. ATCC *hadA* gene; Ap^R^ | This study |
| pMtrcFldH | pMtrc9 derivative; *trc* promoter, the *C. botulinum* A str. ATCC 3502 *fldH* gene; Ap^R^ | This study |

^a^Stratagene, La Jolla, CA.

^b^Invitrogen, Carlsbad, CA.

^c^Novagen, Darmstadt, Germany.

**Supplementary Table 4.** Primers used in this study

| **Primers** | **Sequences** |
| --- | --- |
| Pcthis-F | CGCGCATATGAGAAAGGTTCCCATTATTAC |
| Pcthis-R | CGCGGGATCCTTAGTGATGGTGATGGTGGTGGGACTTCATTTCCTTCAGAC |
| 4CLhis-F | TACAGAATTCATGTTCCGCAGCGAGTACGC |
| 4CLhis-R | TATTCCTGCAGGTTAGTGATGGTGATGGTGGTGTCGCGGCTCCCTGAGCTGTC |
| 4CLmut-F | TACATCGTCAGCGGCGCC |
| 4CLmut-R | GGCGCCGCTGACGATGTA |
| FldAhis-F | CGCGCATATGGAAAACAATGCAAACATGTT |
| FldAhis-R | CGCGAAGCTTTTAGTGATGGTGATGGTGGTGTTTTTCTTTGCGAACCATGATA |
| AroG-F | CGCGGAATTCATGAATTATCAGAACGACGA |
| AroG-R | TATTAAGCTTTTACCCGCGACGCGCTTTTA |
| PheA-F | TATCAAGCTTACACAGGAAACAGAAATGACATCGGAAAACCCGTT |
| PheA-R | CGCGAAGCTTTCAGGTTGGATCAACAGGCA |
| PheAmut-F | ACAATCTGATTATGCCCCGTCTGGAATCAC |
| PheAmut-R | GTGATTCCAGACGGGGCATAATCAGATTGT |
| PAL-Hin-F | GCGCAAGCTTACACAGGAAACAGAAATGGGGACCTTCGTTATTGA |
| PAL-Hin-R | CGCGAAGCTTTTATCACTTGTCATCGTCAT |
| FldH-F | TATAGGATCCATGAAAATCCTGGCGTATTGCG |
| FldH-R | CGCGAAGCTTTTATTTACAAACGCGCTGGT |
| Trc-F | CGCGCTCGAGGCTGTTGACAATTAATCATC |
| Ter-R | CGCGGAGCTCTGTAGAAACGCAAAAAGGCC |
| FldA-F | TATACCTGCAGGACACAGGAAACAGAAATGGAAAACAATGCAAACAT |
| FldA-R | TATGCCTGCAGGTTAGTGATGGTGATGGTGGT |
| FldH-sbF | TATACCTGCAGGACACAGGAAACAGAAATGAAAATCCTGGCGTATTGCG |
| FldH-hiR | CGCGAAGCTTTTATTTACAAACGCGCTGGT |
| HadA-hisF | CGGCCATATGCTTTTAGAAGGAGTTAAAGT |
| HadA-hisR | TATTGCGGCCGCTTAGTGATGGTGATGGTGGTGATATCTTACAACTTTACTAT |
| HadA-sbF | TATTCCTGCAGGCGGATAACAATTTCACACAGGAAACAGAATTCATGCTTTTAGAAGGAGTTAA |
| HadA-ndR | CGCGCATATGTTAATATCTTACAACTTTAC |
| HadA-sbmF | TATTCCTGCAGGACACAGGAAACAGAAATGCTTTTAGAAGGAGTTAA |
| HadA-sbmR | TATACCTGCAGGTTAATATCTTACAACTTTAC |
| PhaAB-BamF | TATAGGATCCCGGGCAAGTACCTTGCCGAC |
| PhaAB-sbR | TATCAAGCTTTCAGCCCATATGCAGGCCGC |
| 100-Kpn-F | TATTGGTACCTTGACGGCTAGCTCAGTCCTAGGTACAGTGCTAGCGAATTCACAGGAAACAGACCATGACTGACGTTGTCATCGT |
| PhaB-Bam-R | TATTGGATCCTCAGCCCATATGCAGGCCGC |
| 105-Kpn-F | TATTGGTACCTTTACGGCTAGCTCAGTCCTAGGTACTATGCTAGCGAATTCACAGGAAACAGACCATGACTGACGTTGTCATCGT |
| 114-Kpn-F | TATTGGTACCTTTATGGCTAGCTCAGTCCTAGGTACAATGCTAGCGAATTCACAGGAAACAGACCATGACTGACGTTGTCATCGT |
| 109-Kpn-F | TATTGGTACCTTTACAGCTAGCTCAGTCCTAGGGACTGTGCTAGCGAATTCACAGGAAACAGACCATGACTGACGTTGTCATCGT |
| 103-Kpn-F | TATTGGTACCCTGATAGCTAGCTCAGTCCTAGGGATTATGCTAGCGAATTCACAGGAAACAGACCATGACTGACGTTGTCATCGT |
| tyrRKO-F | ATAGTGTCATATCATCATATTAATTGTTCTTTTTTCAGGTGAAGGTTCCCTAGGTGACACTATAGAACGCG |
| tyrRKO-R | CGGCTGGTGATTTCGTCCAGCGAACCTTCCATCGCATCTTCGCCCACGGCTAGTGGATCTGATGGGTACC |
| tyrRKO-EXF | TTTCCGTCTTTGTGTCAATGATTGTTGACAGAAACCTTCCTGCTATCCAAATAGTGTCATATCATCATAT |
| tyrRKO-EXR | GCGTGCTGGGATAATTGCGATAAAGCTGGGTTAATACCGAGCGTTCAAAACGGCTGGTGATTTCGTCCAG |
| poxBKO-F | TTTCTCTCCCATCCCTTCCCCCTCCGTCAGATGAACTAAACTTGTTACCGGACACTATAGAACGCGGCCG |
| poxBKO-R | GCGCAGCATATACAGGCTGAAACCTTTGGCCTGTTCGAGTTTGATCTGCGCCGCATAGGCCACTAGTGGA |
| poxBKO-EXF | TATGCCCGATGATATTCCTTTCATCGGGCTATTTAACCGTTAGTGCCTCCTTTCTCTCCCATCCCTTCCC |
| poxBKO-EXR | TTTGTTTTCGCCAGTTCGATCACTTCATCACCGCGTCCGCTGATGATTGCGCGCAGCATATACAGGCTGA |
| pflBKO-F | TACCAAAGGTGACTGGCAGAATGAAGTAAACGTCCGTGACTTCATTCAGAGACACTATAGAACGCGGCCG |
| pflBKO-R | GCGAGTTGAAACGTACTGCGTAGCCAGATACACGGATGGTCAGCTGCGGACCGCATAGGCCACTAGTGGA |
| pflBKO-EXF | TGTTACATGTCCGAGCTTAATGAAAAGTTAGCCACAGCCTGGGAAGGTTTTACCAAAGGTGACTGGCAGA |
| pflBKO-EXR | AGATTGAGTGAAGGTACGAGTAATAACGTCCTGCTGCTGTTCTTTAGTCAGCGAGTTGAAACGTACTGCG |
| adhEKO-F | TGAACTTAACGCACTCGTAGAGCGTGTAAAAAAAGCCCAGCGTGAATATGGACACTATAGAACGCGGCCG |
| adhEKO-R | GCTTTTTTCTCAGCTTTAGCCGGAGCAGCTTCTTTCTTCGCTGCAGTTTCCCGCATAGGCCACTAGTGGA |
| adhEKO-EXF | AAAAAAGTTTAACATTATCAGGAGAGCATTATGGCTGTTACTAATGTCGCTGAACTTAACGCACTCGTAGAG |
| adhEKO-EXR | AGGGGCCGTTTATGTTGCCAGACAGCGCTACTGATTAAGCGGATTTTTTCGCTTTTTTCTCAGCTTTAGCCG |
| frdBKO-F | GCGGAAGCAGCCAATAAGAAGGAGAAGGCGAATGGCTGAGATGAAAAACCGACACTATAGAACGCGGCCG |
| frdBKO-R | GACGTGTTTCGGGCAGACTTCGGAGCAGTAGCCCACGAAAGTACAGCTCCCCGCATAGGCCACTAGTGGA |
| frdBKO-EXF | TGCCGCCAGCTAAACGCGTTTACGGTGGCGAAGCGGATGCAGCCGATAAGGCGGAAGCAGCCAATAAGAA |
| frdBKO-EXR | AAGTCTTTCGAACTTTCTACTTTGCCCTGCTGAATGGCCGCAGCCGGATCGACGTGTTTCGGGCAGACTT |
| tyrBKO-F | GTGTTTCAAAAAGTTGACGCCTACGCTGGCGACCCGATTCTTACGCTTATTAGGTGACACTATAGAACGCG |
| tyrBKO-R | TGGCAATGGCGCGAATAGCGTAGGCATCCTCTTCCATACCGGCACCAAATTAGTGGATCTGATGGGTACC |
| tyrBKO-EXF | CCGGTTTATTGTGTTTTAACCACCTGCCCGTAAACCTGGAGAACCATCGCGTGTTTCAAAAAGTTGACGC |
| tyrBKO-EXR | GGAGAAAATTTTCGAGAACGAATTGCTCACCAGAGCGGGTAATCCAGCGCTGGCAATGGCGCGAATAGCG |
| aspCKO-F | ATGTTTGAGAACATTACCGCCGCTCCTGCCGACCCGATTCTGGGCCTGGCTAGGTGACACTATAGAACGCG |
| aspCKO-R | TAGCCGCGAAAGCGCGCAGTCCTTCAGCATCTTCTTCCAGACCACGGGCATAGTGGATCTGATGGGTACC |
| aspCKO-EXF | CGTTACCCTGATAGCGGACTTCCCTTCTGTAACCATAATGGAACCTCGTCATGTTTGAGAACATTACCGC |
| aspCKO-EXR | CAGGCCAAAGTTTTTAGAGTAGGAACTGGCAACAATCAGCTCTTTATGCATAGCCGCGAAAGCGCGCAGT |
| ldhAKO-F | ACAGGTGAACGAGTCCTTTGGCTTTGAGCTGGAATTTTTTGACTTTCTGCGACACTATAGAACGCGGCCG |
| ldhAKO-R | TTGCTTAAGTTTTGCAGCGTAGTCTGAGAAATACTGGTCAGAGCTTCTGCCCGCATAGGCCACTAGTGGA |
| ldhAKO-EXF | ATGAAACTCGCCGTTTATAGCACAAAACAGTACGACAAGAAGTACCTGCAACAGGTGAACGAGTCCTTTG |
| ldhAKO-EXR | AGCGGCAAGATTAAACCAGTTCGTTCGGGCAGGTTTCGCCTTTTTCCAGATTGCTTAAGTTTTGCAGCGT |
| ldhArep-R | TTGCTTAAGTTTTGCAGCGTAGTCTGAGAAATACTGGTCAGAGCTTCTGCTGAGCGGATACATATTTGAATGTATTT |

*Restriction sites are underlined.

**Supplementary Note 1: 4CL enzyme assay**

4CL was found to successfully catalyze the conversion of cinnamate into cinnamoyl-CoA (Supplementary Fig. 2a). After 1h reaction, cinnamoyl-CoA was detected by the spectrophotometric assay, as previously reported (Supplementary Fig. 2b, c)^8,9^. The changes in absorbance during cinnamoyl-CoA formation were monitored at wavelengths of 311 nm (cinnamoyl-CoA) and 412 nm (free CoA). The absorbance at 311 nm was increased after 1h reaction. Moreover, mutant 4CL having amino acid change (A294G)^9^ exhibited higher activity toward cinnamate compared to wild-type 4CL (Supplementary Fig. 2b). The formation of cinnamoyl-CoA was also confirmed by LC-MS (Supplementary Fig. 2c). Based on these results, 4CL (A294G) was selected for the synthesis of cinnamoyl-CoA.

**Supplementary Note 2: *In silico* flux response analysis**

To further increase the d-phenyllactic acid production, the responses of the d-phenyllactic acid production rate to the varying intracellular fluxes of central and aromatic amino acid biosynthesis reactions were investigated using *in silico* flux response analysis. The relationships between d-phenyllactic acid production rate and intracellular fluxes were categorized into six types as shown in Supplementary Figure 8. The reactions belonging to negative correlations with the d-phenyllactic acid production rate can be potential knockout candidates. *In silico* flux response analysis demonstrated that d-phenyllactic acid production will be increased by deletion of the negatively correlated reactions belonging to the pentose phosphate pathways and aromatic amino acid biosynthesis pathways. According to the flux response analysis, the *tyrB* and *aspC* genes, which are involved in aromatic amino acid biosynthesis pathway, were selected as knockout targets.

**Supplementary Note 3: Characterization of aromatic PHAs**

The thermal properties of aromatic PHAs with different d-phenyllactate monomer compositions were analysed by differential scanning calorimetry (DSC) (Fig. 4c). In a good agreement with previous studies^10,11^, pristine poly(3HB) exhibited very strong crystalline property as evidenced by a distinctive crystal melting peak at temperature (T_m_) of 165 ^o^C. As the mole fraction of d-phenyllactate increased, both T_m_ values and heat of fusion (ΔH) from the crystalline phases were gradually decreased, indicating that the incorporation of d-phenyllactate greatly reduces the formation of crystalline structures. Their crystalline properties were further examined by grazing incident X-ray scattering (GIXS) (Fig. 4d-h). As the content of d-phenyllactate in the copolymer film increased, prominent scattering peaks from crystalline phase from poly(3HB) gradually decreased. Also, poly(52.1 mol% 3HB-*co*-47.9 mol% d-phenyllactate) film shows only amorphous halos around *q* = 1.2~1.4 Å^-1^. In addition, the glass transition temperature (T_g_) of the copolymers was observed to be greatly increased at the same time as the d-phenyllactate content increased in the copolymers. Such reduced crystallinity and increased T_g_ of the aromatic PHAs, which generally render much higher mechanical toughness to the polymer, make them more competitive candidates for industrial applications, whereas strong stiffness and brittleness of poly(3HB) greatly limit their practical applications. To examine the mechanical properties of the aromatic PHA polymers, the cohesive failure energies of a series of the polymers were compared using the double cantilever beam (DCB) fracture mechanics testing method (Fig. 4i).

**Supplementary Method**

**Characterization of poly(52.3 mol% 3HB-*co*-47.7 mol% d-phenyllactate)**. ^1^H NMR (600 MHz, CDCl_3_): δ 7.25-6.94 (br, C_6_*H*_5_ for PhLA), 5.26-4.91 (br, C*H* for 3HB and C*H* for PhLA), 3.20-2.76 (br, C*H*_2_ for PhLA), 2.60-2.31 (m, C*H*_2_ for 3HB), 1.21-1.07 (m, C*H*_3_ for 3HB); ^13^C NMR (150 MHz, CDCl_3_): δ 169.4-168.1 (*C*O for 3HB and *C*O for PhLA), 136.0-135.4 (*C* of C_6_H_5_ for PhLA), 129.3, 128.4 and 127.1 (*C*Hs of C_6_H_5_ for PhLA), 73.5-72.8 (*C*H for PhLA), 68.8-67.5 (*C*H for 3HB), 40.8-39.9 (*C*H_2_ for 3HB), 37.1-36.9 (*C*H_2_ for PhLA), 19.8-19.4 (*C*H_3_ for 3HB).

**Characterization of poly(16.8 mol% d-lactate-*co*-80.8 3HB-*co*-1.6 mol% d-phenyllactate-*co*-0.8 mol% d-4-hydroxyphenyllactate)**. ^1^H NMR (600 MHz, CDCl_3_): δ 7.23-7.15 (m, C_6_*H*_5_ for PhLA), 6.99 (d, *J* = 8.0 Hz) and 6.70 (d, *J* = 7.6 Hz) (C*H* of 4-HOC_5_H_4_ for 4HPh), 5.19 (sext, *J* = 6.4 Hz, C*H* for 3HB), 5.11-4.87 (m, C*H* for PhLA, C*H* for 4HPh and C*H* for LA), 3.11-2.96 (m, C*H_2_* for PhLA and C*H_2_* for 4HPh), 2.64-2.38 (m, C*H_2_* for 3HB), 1.52-1.36 (m, C*H*_3_ for LA), 1.27-1.15 (m, C*H*_3_ for 3HB).

**Characterization of poly(55.2 mol% 3HB-*co*-43.0 mol% d-phenyllactate-*co*-1.8 mol% d-mandelate)**. ^1^H NMR (600 MHz, CDCl_3_): δ 7.38-6.94 (br, C_6_*H*_5_ for PhLA and C_6_*H*_5_ for MA), 5.97-5.95 (br, C*H* for MA), 3.24-2.82 (br, C*H*_2_ for PhLA), 2.70-2.21 (br, C*H*_2_ for 3HB), 1.26-1.16 (m, C*H*_3_ for 3HB).

**Characterization of poly(33.3 mol% 3HB-*co*-18.0 mol% d-phenyllactate-*co*-48.7 mol% d-3-hydroxy-3-phenylpropionate)**. ^1^H NMR (600 MHz, CDCl_3_): δ 7.26-6.96 (br, C_6_*H*_5_ for PhLA and C_6_*H*_5_ for 3HPh), 6.12-5.90 (br, C*H* for 3HPh), 5.25-4.91 (br, C*H* for 3HB and C*H* for PhLA), 3.16-2.16 (br, C*H*_2_ for 3HB, C*H*_2_ for PhLA, and C*H*_2_ for 3HPh), 1.25-0.90 (br, C*H*_3_ for 3HB); ^13^C NMR (150 MHz, CDCl_3_): δ 169.2-168.4 (*C*O for 3HB, *C*O for PhLA, and *C*O for 3HPh), 139.0-138.9 (*C* of C_6_H_5_ for 3HPh), 136.1 (*C* of C_6_H_5_ for PhLA), 129.5-126.4 (*C*Hs of C_6_H_5_ for PhLA and *C*Hs of C_6_H_5_ for 3HPh), 72.6-72.4 (*C*H for PhLA and *C*H for PhLA), 67.9-67.7 (*C*H for 3HB), 41.2-40.3 (*C*H_2_ for 3HB and *C*H_2_ for 3HPh), 37.1 (*C*H_2_ for PhLA), 19.7-19.6 (*C*H_3_ or 3HB).

**Mass data of mandelyl-CoA**. LC/MSD-ESI (*m*/*z*): [M-H]^-^ calcd for C_29_H_41_N_7_O_18_P_3_S, 900.1442; found 900.3.

**Mass data of 3-hydroxy-3-phenylpropionyl-Co**. LC/MSD-ESI (*m*/*z*): [M-H]^-^ calcd for C_30_H_43_N_7_O_18_P_3_S, 914.1598; found 914.3.

**Mass data of 2-hydroxy-4-phenylbutyryl-CoA**. LC/MSD-ESI (*m*/*z*): [M-H]^-^ calcd for C_31_H_45_N_7_O_18_P_3_S, 828.1755; found 828.3.

**Mass data of 4-phenylbenzoyl-CoA**. LC/MSD-ESI (*m*/*z*): [M-H]^-^ calcd for C_28_H_39_N_7_O_18_P_3_S, 886.1285; found 886.3.

**Mass data of acetyl-CoA**. LC/MSD-ESI (*m*/*z*): [M-H]^-^ calcd for C_23_H_37_N_7_O_17_P_3_S, 808.1179; found 808.1.

**Mass data of cinnamoyl-CoA**. LC/MSD-ESI (*m*/*z*): [M-H]^-^ calcd for C_30_H_41_N_7_O_17_P_3_S, 896.1492; found 896.2.

**Mass data of phenyllactyl-CoA**. LC/MSD-ESI (*m*/*z*): [M-H]^-^ calcd for C_30_H_43_N_7_O_18_P_3_S, 914.1598; found 914.0.

**Mass data of 4-hydroxyphenyllactyl-CoA**. LC/MSD-ESI (*m*/*z*): [M-H]^-^ calcd for C_30_H_43_N_7_O_19_P_3_S, 930.1547; found 930.0.

**Mass data of 2-hydroxyisocaproyl-CoA**. LC/MSD-ESI (*m*/*z*): [M-H]^-^ calcd for C_27_H_45_N_7_O_18_P_3_S, 880.1755; found 880.3.

**Mass data of propionyl-CoA**. LC/MSD-ESI (*m*/*z*): [M-H]^-^ calcd for C_24_H_39_N_7_O_17_P_3_S, 822.1336; found 822.3.

**Mass data of butyryl-CoA**. LC/MSD-ESI (*m*/*z*): [M-H]^-^ calcd for C_25_H_41_N_7_O_17_P_3_S, 836.1493; found 836.3.

**Mass data of lactyl-CoA**. LC/MSD-ESI (*m*/*z*): [M-H]^-^ calcd for C_24_H_39_N_7_O_18_P_3_S, 838.1285; found 838.3.

**Mass data of 2-hydroxybutyryl-CoA**. LC/MSD-ESI (*m*/*z*): [M-H]^-^ calcd for C_25_H_41_N_7_O_18_P_3_S, 852.1442; found 852.3.

**Mass data of 3-hydroxybutyryl-CoA**. LC/MSD-ESI (*m*/*z*): [M-H]^-^ calcd for C_25_H_41_N_7_O_18_P_3_S, 852.1442; found 852.3.

**Mass data of 4-hydroxybutyryl-CoA**. LC/MSD-ESI (*m*/*z*): [M-H]^-^ calcd for C_25_H_41_N_7_O_18_P_3_S, 852.1442; found 852.3.

**Mass data of 5-hydroxyvaleryl-CoA**. LC/MSD-ESI (*m*/*z*): [M-H]^-^ calcd for C_26_H_43_N_7_O_18_P_3_S, 866.1598; found 866.3.

**Mass data of 6-hydroxyhexanoyl-CoA**. LC/MSD-ESI (*m*/*z*): [M-H]^-^ calcd for C_27_H_45_N_7_O_18_P_3_S, 880.1755; found 880.3.

**SUPPLEMENTARY REFERENCES**

1. Datsenko, K. A. & Wanner, B. L. One-step inactivation of chromosomal genes in *Escherichia coli* K-12 using PCR products. *Proc. Natl Acad. Sci. USA* **97**, 6640-6645 (2000).

2. Lee, K. H., Park, J. H., Kim, T. Y., Kim, H. U. & Lee, S. Y. Systems metabolic engineering of *Escherichia coli* for L-threonine production. *Mol. Syst. Biol.* **3**, 149 (2007).

3. Palmeros, B. *et al.* A family of removable cassettes designed to obtain antibiotic-resistance-free genomic modifications of *Escherichia coli* and other bacteria. *Gene* **247**, 255-264 (2000).

4. Park, S. J. *et al.* Metabolic engineering of *Ralstonia eutropha* for the biosynthesis of 2-hydroxyacid-containing polyhydroxyalkanoates. *Metab. Eng.* **20**, 20-28 (2013).

5. Yang, T. H. *et al.* Biosynthesis of polylactic acid and its copolymers using evolved propionate CoA transferase and PHA synthase. *Biotechnol. Bioeng.* **105**, 150-160 (2010).

6. Yang, T. H. *et al.* Tailor-made type II *Pseudomonas* PHA synthases and their use for the biosynthesis of polylactic acid and its copolymer in recombinant *Escherichia coli*. *Appl. Microbiol. Biotechnol.* **90**, 603-614 (2011).

7. Choi, S. Y. *et al.* One-step fermentative production of poly(lactate-*co*-glycolate) from carbohydrates in *Escherichia coli*. *Nat. Biotechnol.* **34**, 435-440 (2016).

8. Knobloch, K. H. & Hahlbrock, K. 4-Coumarate:CoA ligase from cell suspension cultures of *Petroselinum hortense* Hoffm. Partial purification, substrate specificity, and further properties. *Arch. Biochem. Biophys.* **184**, 237-248 (1977).

9. Kaneko, M., Ohnishi, Y. & Horinouchi, S. Cinnamate:coenzyme A ligase from the filamentous bacterium *streptomyces coelicolor* A3(2). *J. Bacteriol.* **185**, 20-27 (2003).

10. Abe, H., & Doi, Y. Side-chain effect of second monomer units on crystalline morphology, thermal properties, and enzymatic degradability for random copolyesters of (R)-3-hydroxybutyric acid with (R)-3-hydroxyalkanoic acids. *Biomacromolecules* **3**, 133-138 (2002).

11. Satoh, H., Yoshie, N., & Inoue, Y. Hydrolytic degradation of blends of poly (3-hydroxybutyrate) with poly (3-hydroxybutyrate-*co*-3-hydroxyvalerate). *Polymer* **35**, 286-290 (1994).
